# On psychosocial pathomorphosis of depression

**DOI:** 10.1192/j.eurpsy.2021.881

**Published:** 2021-08-13

**Authors:** V. Krasnov, N. Semenova

**Affiliations:** Psychiatry, Moscow Research Institute of Psychiatry – a branch of V.Serbsky National Medical Research Centre for Psychiatry and Narcology, Moscow, Russia, Moscow, Russian Federation

**Keywords:** Depression, Psychosocial pathomorphosis, Patient’s capacity to verbalize feelings

## Abstract

**Introduction:**

The concept of depression has long been a matter of controversy. Sociocultural factors greatly influence the phenomenology of depression and the meaning that patients assign to their symptoms.

**Objectives:**

The aim is to determine the changes in the phenomenology of depression over the past decades.

**Methods:**

To compare the proportions of biologically mediated symptoms of typical recurrent melancholic depression with the ideator components of the depressive syndrome and a depressive decrease in reactivity. We compared the archival data of one of the authors (V.N.K.) obtained in the study of depression: 1980-1987 (first group) and 2014-2020 (second group). The groups are age-comparable (21-64 y.o.). The Hamilton Depression Scale has been used to assess depression (score of 21–32, in both groups).

**Results:**

Basic, i.e., biologically mediated symptoms, were not statistically different in the study groups. Whereas symptoms associated with emotional reactivity, the patient’s introspective abilities and capacity to identify and verbalize feelings - in the second group, were statistically rare, except for anhedonia, which, on the contrary, came to the fore. Based on some longitudinal studies of the dangers of excessive reliance on computer-mediated communication, one could foresee such contrasting phenomenology changes, which were especially clearly manifested in young patients.

**Conclusions:**

Over the past decades, there are changes in the phenomenology of depression. The same underlying disorder can produce different clinical presentations, and agreement on a pathological entity does not necessarily mean deal with a descriptive label.

